# Structured lactation support and human donor milk for German NICUs—Protocol on an intervention design based on a multidimensional status quo and needs assessment (Neo-MILK)

**DOI:** 10.1371/journal.pone.0284621

**Published:** 2023-04-27

**Authors:** Nadine Scholten, Alicia Fitzgerald, Katja Matthias, Mi-Ran Okumu, Tim Ohnhäuser, Katharina Schmitz, Christine Schreiner, Isabella Schwab, Anna Stirner, Ricarda Wullenkord, Till Dresbach, on behalf of Neo-MILK

**Affiliations:** 1 Faculty of Medicine and University Hospital Cologne, Institute of Medical Sociology, Health Services Research, and Rehabilitation Science, Chair for Health Services Research, University of Cologne, Cologne, Germany; 2 Faculty of Law, Henrich Heine University Duesseldorf, Düsseldorf, Germany; 3 TAKEPART Media + Science GmbH, Cologne, Germany; 4 Division of Neonatology, Center for Pediatric and Adolescent Medicine, University Medical Center of the Johannes Gutenberg-University Mainz, Mainz, Germany; 5 Dept. of Neonatology and Pediatric Intensive Care, University Hospital Bonn, Bonn, Germany; 6 Faculty of Management, Economics and Social Sciences, Department of Business Administration and Health Care Management, University of Cologne, Cologne, Germany; 7 CITEC, Universität Bielefeld, Bielefeld, Germany; Public Library of Science, UNITED KINGDOM

## Abstract

**Introduction:**

Mother’s own milk is the best nutrition for every newborn and especially for vulnerable infants such as preterm infants with a very low birth weight below 1,500 grams (VLBW). If no MOM is available, human donor milk is the alternative of choice. Mothers of preterm born infants face challenging conditions that impair sufficient milk production. For this reason, it is particularly important to provide structural lactation support and, at the same time, to promote the establishment of human donor milk banks.

**Methods and analysis:**

Via a multidisciplinary approach the Neo-MILK study will develop an intervention for structured breastfeeding and lactation support. This will be based on a comprehensive status quo and needs assessment. In addition, the implementation of human donor milk banks (HDMB) will be supported by the development of standards.

**Ethics and dissemination:**

Intervention development is participatory, involving different disciplines and stakeholders. All surveys are subject to approval by the ethics committee. During the course of the project, the results will be communicated to the scientific community and the general public via publications, the project homepage and social media.

**Trial registration number:**

DRKS00024799 (German Clinical Trials Register).

## Introduction

Mother’s own milk (MOM) is the best nutrition for every newborn. For more than 40 years, the World Health Organization (WHO) has recommended exclusive feeding with MOM from the first day of life on [1, 2]. Optimal nutrition with human milk (HM) for vulnerable newborns, such as preterm infants with a very low birth weight below 1,500 grams (VLBW) or newborns with congenital diseases, is particularly crucial regarding mortality and their further development [3–7]. VLBW infants fed exclusively with MOM have a lower risk of necrotising enterocolitis (NEC) [8–11], late onset sepsis (LOS) [12], retinopathy of prematurity (ROP) [13, 14] and bronchopulmonary dysplasia (BPD), a faster food build-up, as well as improved neurologic outcomes [15, 16]. If MOM (number one choice of nutrition) is not available at all or temporarily unavailable, human donor milk HDM is the alternative nutrition of choice, prior to the use of artificial nutrition (formula) for VLBW infants [17, 18]. This is based, on the one hand, on the clinical benefits in terms of infant health outcomes (significantly lower NEC rate for HDM in combination with MOM [19] and, on the other hand, the negative effects of formula feeding on breastfeeding/MOM supply that have been shown [20]. Studies confirming the positive impact of the presence of a human donor milk bank (HDMB) on the rate of MOM fed children at discharge emphasise the processes and changes that the establishment and operation of a HDMB entails in a clinic, like an increased focus by staff and parents on the benefits of HM nutrition [21, 22].

Even though secretory differentiation (former lactogenesis I) happens by mid pregnancy [23] and thus successful lactation is possible despite prematurity, this complex physiological process is negatively affected at different levels by prematurity. Due to the immaturity of the preterm infant, it is necessary to initiate and maintain milk production in a timely manner after birth via mechanical pumping or by manual emptying of the breast instead of direct feeding at the breast [24, 25]. The separation of mother and child, as well as the psychological stress induced by the circumstances of preterm birth, can additionally impair lactation. Therefore, a structured promotion of lactation is particularly necessary for mothers of preterm infants [26, 27]. If breastfeeding intention and lactation are supported in a structured manner, a large proportion of infants in neonatal intensive care units (NICUs) can be fed with MOM immediately or after a short period of time. Evidence-based interventions to increase the rate of preterm infants fed with MOM include, for example, staff and parent education on early initiation of lactation, maintenance of lactation, handling of HM, breastfeeding skills of the preterm, and transfer to successful breastfeeding [28, 29].

Currently, there is no structured care for mothers of preterm infants in Germany with regard to lactation and breastfeeding [30]. At the same time, HDMBs are currently implemented in only a few NICUs in Germany [31]. The fact that HDM is not currently available in all NICUs in Germany is partly due to the unclear legal nature of DM in Germany, as well as the currently outdated guidelines on the operation of an HDMB [31].

### Aims of the study

**Fig 1:**
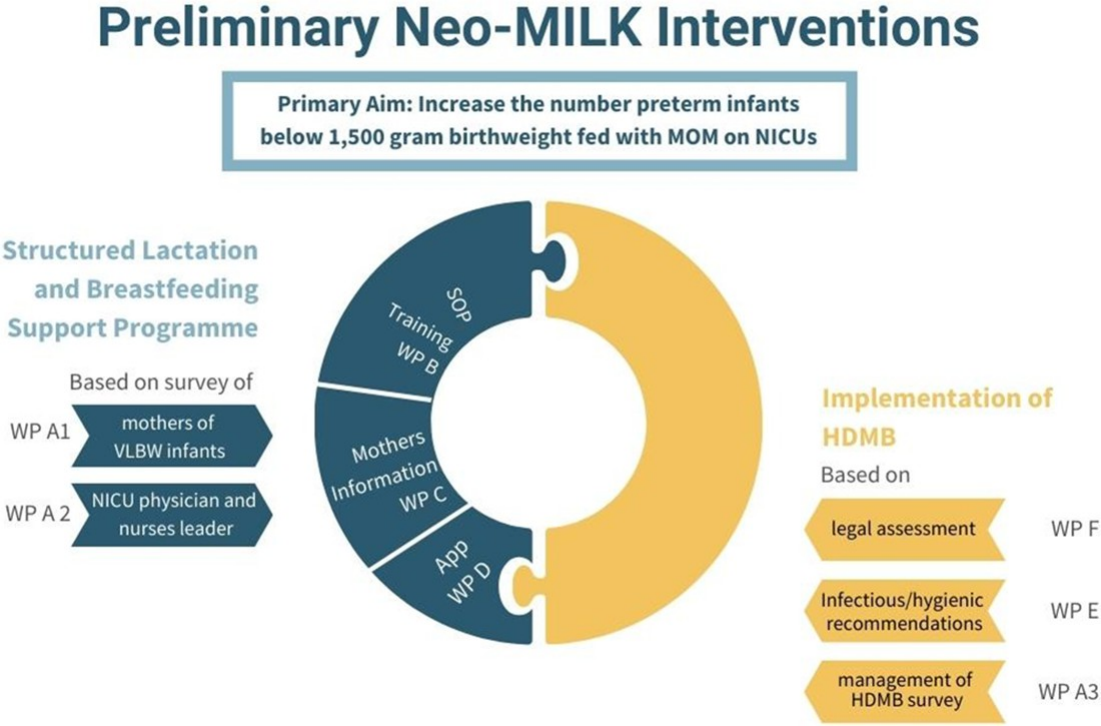
Structure of the Neo-MILK intervention based on the work packages (WP).

The aim of the Neo-MILK study is to develop a structured lactation and breastfeeding support programme, explicitly adapted to the needs of parents of preterm infants weighing less than 1,500 grams in the German NICU setting. The structured breastfeeding and lactation programme will be accompanied by an app, which will be designed to support mothers during the pumping period. This will be by provision of evidence-based information (videos and text) and by reminding mothers to pump regularly and at the same time helping to document the amount of expressed milk. The structured lactation support will be complemented by the establishment of HDMBs, which also aim to achieve the primary study goal of ensuring that as many VLBW preterm infants as possible are fed with MOM (Fig 1).

The lactation and breastfeeding support programme will include the following preliminary elements, which are based on the 10 steps of the WHO’s baby-friendly hospital initiative (BFHI) [32] and supplemented by further specific measures with international evidence [33, 34] and explicitly adapted to the target group (preterm infants under 1,500 grams) and setting (German NICU).

For this purpose, it is essential to learn more about the status quo of care, as well as the actual needs of mothers and caregivers (NICU physicians and nurses). The breastfeeding and lactation support programme, as well as the HDMB implementation concept will be based on the following preliminary WP. Wherever necessary, additional ad hoc surveys will be conducted to further the development of the intervention package.

Essential to the development of the standards for the implementation of the HDMB are the legal report (WP E) to be prepared as part of the project, as well as the infectious/hygienic recommendations (WP F).

In addition to the development of the intervention, the implementation of the intervention in 15 NICUs, as well as the evaluation of this intervention will take place within the scope of the Neo-MILK project.

## Materials and methods

The development of the intervention will be based on mixed-methods (qualitative and quantitative) surveys considering the different circumstances of the diverse target groups (mothers of VLBW infants, senior physicians/nurses, HDMB management).

### Work package A1: Mothers of preterm infants under 1,500 grams

The mixed-methods survey of mothers will follow a sequential exploratory design.

#### Work package A.1.1. Structured qualitative interviews

At least 12 interviews are planned with mothers of VLBW infants under 1,500 grams in the 3–12 months after hospital discharge of the children. This time period was chosen in trade-off between retraumatisation risk and recall reliability. Mothers who gave birth to multiples of which not all infants survived are excluded. Due to the Covid-19 pandemic, interviews will be conducted remotely. As the target group being German mothers of VLBW infants, the interview language will be German. Answers will be captured using an offline dictation machine. To reduce the risk of data loss due to malfunctioning equipment, a transcript writer will be present if the mothers provide consent. Interviews will be done in a semi-structured manner to provide the option to ask further questions and/or comfort the mothers if they show signs of psychological stress, and will be conducted by a psychologist to further mitigate retraumatisation risk.

Based on the results of a literature research the interview guide will cover the following topics:

- Breastfeeding intention.
- Current breastfeeding and pumping behaviour.
- Breastfeeding and pumping attitudes and related norms.
- Stay at NICU (e.g. framework data, breastfeeding and pumping behaviour during the stay).
- Lactation and breastfeeding support (e.g. by hospital/NICU staff, partner etc.).
- Psychological stress factors.
- Gender role orientation.
- Previous breastfeeding and pumping experience and behaviour
- Breastfeeding app opinions and preferences.
- Demographic questions (e.g. age, vocational status, religion etc.).

Questions will be developed by experts and based on the topics of the literature review, and will be discussed and revised within a multidisciplinary team including psychologists, social scientist, medical experts (especially with expertise in NICU), nursing staff, as well as behavioural economists. Once the interviews are conducted, the resulting audio files will be transcribed and anonymised by a transcription office and will be coded by two trained research assistants according to a coding scheme. Interview data will be analysed qualitatively and, where possible, data will be coded in a way to also enable quantitative analyses.

#### Work package A.1.2. Anonymous quantitative cross-sectional survey

Based on the current literature and the qualitative interviews (Work package A.1.1), a paper–pencil questionnaire will be developed to assess the current status of breastfeeding and lactation support from the perspective of the mothers concerned.

Topics to be covered by the questionnaire include:

- How was breastfeeding and lactation support experienced?
- How was lactation initiated and maintained?
- How was/were the infant(s) fed in the NICU?
- What is the attitude towards milk donation, as well as the acceptance of HDM?

Where possible, validated instruments will be used. If no suitable instruments are available, questions developed in-house will be applied. This will involve a multidisciplinary team consisting of, among others, social scientists, sociologists, and physicians. The questionnaire will be pre-tested by the relevant target group before implementation and will be sent out by the cooperating health insurance companies. In Germany, everyone is obliged to have health insurance. For this survey, four statutory health insurance companies with a market share of 28% are cooperating. Mothers insured by the participating health insurance companies whose infants are between six and 18 months old at the time of the survey will be invited to participate. Identification in the health insurance data will be by the following ICD-10 codes: P07.01, P07.02, P07.10 and P07.11. The expected sample size is approximately 2,000, whereof a response rate of approximately 30% is expected. An unconditional incentive (plaster set and gummy bears) will be enclosed with the survey documents. At the same time, every participant will have the opportunity to take part in a raffle for gift vouchers and small prizes. The anonymous questionnaires will be automatically read and processed for further statistical evaluation. Descriptive and multivariate analytical evaluations will be carried out.

### Work package A.2: Head physicians/Head nurses of the NICU

#### Quantitative survey

Execution of an anonymous Germany-wide postal survey of all Level I and Level II NICU leaders (n = 211 physicians and n = 211 nurses).

Topics to be covered by the questionnaire include:

- What is the perceived value of feeding with MOM and with HDM?
- How does breastfeeding and lactation support take place?
- Is HDM already being given at present?
- What are the reasons for HDMB not being implemented?
- Evaluation of implementation barriers and facilitators.

Where possible, validated instruments will be used. If no suitable instruments are available, questions developed in-house will be applied. This will involve a multidisciplinary team consisting of, among others, social scientists and physicians. The questionnaire will be pre-tested by the relevant target group before implementation. The survey of the physician and nurse NICU leaders will be done anonymously with a response control, so that following Dillmann [36] they can be reminded to participate up to three times. The focus is on identifying structural NICU characteristics that can be considered in intervention development and can lead to tailoring of the intervention there. The survey will be a full survey, and a response rate of at least 65% is expected. Participation in the survey will only be encouraged by the addition of sweets. The aim is to generate a high level of self-motivation through the design of the survey instrument. The anonymous questionnaires will be automatically read and processed for further statistical evaluation. Descriptive and multivariate analytical evaluations will be carried out.

### Work package A.3: Head of HDMB in Germany

The mixed-methods survey of HDMB will follow a sequential explanatory design.

#### Work package A.3.1. Quantitative survey

To capture the current processes, the 34 known HDMBs in Germany will be invited to participate in the anonymous quantitative online survey. In this structured survey, the practice of HDMB will be queried including the individual milk processing steps, recruitment of donors, as well as the perceived implementation hurdles and problems queried. At the end of the online survey, the respondents will be asked whether they are willing to participate in an interview.

#### Work package A 3.2. Structured qualitative interviews

A total of at least six qualitative interviews will be conducted in order to deepen the findings from the quantitative survey and to gain further insights. Interview participants will be selected in a purposeful manner based on the information provided by the survey in order to cover a broad sample. Depending on the wishes of the interview participants, the interviews will be conducted in person, by telephone or by video conference. The interviews will be recorded, transcribed and analysed through content analysis. The structured interview guide will be created based on the structured survey and individually adapted to the interview participant based on the results of the survey.

### Work package B: Standard operating procedure (SOP) and educational training

We will develop a concept to support lactation and breastfeeding, adjusted for the NICU setting and preterm born VLBW infants. In addition, a review of current literature will be carried out to underline and adjust each of the steps indicated by the BFHI for the NICU setting by recent findings.

The concept aims at: (i) the education and training of staff (WP B); and (ii) the provision of information to mothers at risk of having a prematurely born infant and to their partners (WP C). For this purpose, suitable methods and materials will be developed by expert researchers and iteratively agreed upon in a multi-professional team. As a main source to operationalise the breastfeeding and lactation support a comprehensive manual will be required, which will be the reference for education and training on HM, feeding and lactation of both staff members and mothers (and their partners). The first part of the manual will contain exhaustive information on HM, breastfeeding and lactation to support staff members during the (prepartal) education of mothers (parents). Therefore, evidence-based methods for lactation and breastfeeding support for mothers of preterm born infants will be presented. In the second part, the manual will compile methods for the supervision of mothers (parents) during their infants’ stay at the NICU and beyond. The development of the manual will be carried out by medical staff experienced in neonatology, under consideration of input from psychology, behavioural economics as well as pedagogy regarding how to ideally present content for maximal clarity and understanding.

With the manual as a basis, a comprehensive concept for training and education of staff members will be developed. To identify comprehensive key areas of training, different perspectives will be harmonised in the aforementioned multi-professional team. Based on a cooperative effort, several approaches and corresponding materials for educational purposes for staff of NICUs, delivery rooms [32] and gynaecological wards will be devised.

Drawing on presentation-based educational videos, the Neo-MILK staff training will be designed to provide comprehensive education for health professionals on the anatomy and (patho-)physiology of lactation: (i) to achieve a uniform, high standard of knowledge and quality in the handling of HM; and (ii) to foster counselling and support for the mothers and their partners within the NICU. The training will consist of seven detailed modules and a condensed crash course module. It will be offered on a voluntary basis with videos being provided as an on-demand stream via a restricted area on the Neo-MILK project homepage. To obtain visibility among staff and to create an incentive to complete the training, a certificate for participating staff will be offered. To obtain the certificate on successful participation in the training, staff members need to complete a short quiz on the course contents. Furthermore, staff members who successfully participate in training will have the opportunity to be publicly recognised on a poster. Developed on empirical evidence on the effects of public recognition [37], the poster will provide space for the pictures and names of staff members and aims to promote participation in training among staff members. Furthermore, it will contain a slogan to establish the promotion of feeding of premature infants with HM as ward culture and to communicate these efforts made by the staff transparently among other staff members and parents. The development of methods for staff training will be jointly conducted by experts from the fields of medicine, psychology and behavioural economics.

In addition, further educational material such as illustrated informational posters for the NICU and gynaecological wards (e.g. ‘One Minute Wonder’ on initiation and maintenance of lactation) or ‘pocket knowledge’ flyers will be provided.

### Work package C: Information for mothers, i.e. parents, prepartal consultation

For the purpose of informing mothers and their partners, a multi-tool intervention-package will be developed. The package will contain checklists for the prepartal consultation between mothers, their partners, and staff member of the neonatal team (e.g. doctors, nurses or midwives) who are responsible for treatment of VLBW infant(s) and/or their mothers in the case of a premature birth. The checklist for medical staff will be based on the WHO Surgery Checklist [38] to enable and ensure the provision of standardised, evidence-based information about HM, breastfeeding, and lactation. This will be provided as a form containing the option to be signed by all parties participating in the consultation. Thus, it is intended to support the documentation of the consultation as well as being a compliance-promoting, transparent commitment with regard to the contents of discussion and their implementation. The checklist for mothers will be designed as a question prompt sheet (QPS), covering prominent topics such as lactation in prematurity, bonding, and hygienic handling of expressed milk in correspondence with the information provided during prepartal consultation. The QPS is intended to serve as a preparation tool for parents for the prepartal consultation as well as a reference point for later questions or to refresh knowledge gained during the consultation. The development of checklists will be led by experts in behavioural economics.

To support mothers and their partners in transferring received information into practice, ‘mom bags’ will be assembled, which will include equipment, e.g., syringes for collecting colostrum and several flyers with different informational focal points on lactation initiation and maintenance.

The designed material will be pre-tested in a convenience sample population of mothers of VLBW preterm infants recruited within relevant groups of social media platforms and will be adjusted upon feedback by the target group (mothers who gave birth to a preterm or mothers whose infant was treated in a NICU).

### Work package D: App

Together with a media agency, a progressive web application (app) is to be developed that will enable mothers to document their milk volume and to be reminded of pumping times. evidence-based information on the topics of NICU, pumping and HM will be embedded in the app. Unlike existing apps, this tool will specifically target mothers/parents of preterms. It will be developed in an interdisciplinary team consisting of researchers, content editors, designers, and programmers, based on the needs and wishes identified in WP. A. The development of the app will include various work steps. The first one will involve research into existing tools, including their approach, functionality, content and design. Based on the findings of the research and the investigations of the Neo-MILK project, in the course of the user experience design personas will be created, which will provide insights into possible usage scenarios of the app. This will be followed by the conception of the app and the structuring of the content. This process will be recorded and documented in wireframes which will then be used to create an interactive prototype. The content conception of the app will also include the definition of data points to be collected and the development of a concept for handling these data in the context of the research project. Parallel to the content and app development, the elaboration of the design will occur in a multi-stage process. The app will be reflected and designed in a participatory manner at different points in time and will be pre-tested with the target group after completion of the interactive prototype.

### Work package E: Legal regulations

Currently, the classification, i.e., the legal nature of human donor milk is unclear in Germany. There are neither normative regulations nor supreme court decisions on this. Overall, it will be necessary to clarify which basic legal obligations exist for the production of safe HM and its administration within hospitals. In particular, it should be investigated whether and how supervisory authorities are to be involved in the process of donating breast milk, and whether registration, notification, approval, verification or even licensing procedures and obligations are to be fulfilled prior to the first administration of human donor milk in a clinic. In the next step, the basic outlines of the obligations for donor milk banks resulting from the identified regimes will be outlined and elaborated. Based on these findings, we will examine whether the regime opened up by the legal nature is capable of conclusively regulating human donor milk or whether there is a need for supplementation through further development of the law. The (future) legal fate as well as the need for legal regulation of the donation of HM largely depend on the current legislative developments at the EU level. In July 2022, the European Commission presented a draft of an extensive regulation on substances of human origin (COM/2022/338 final), which also includes the donation of HM. It remains to be seen whether this regulation will come into force.

### Work package F: Infectious/hygienic recommendations for HDMB in Germany

Based on the current evidence and German legal requirements, a manual for HDMB will be developed. The manual will offer practice-oriented, scientifically founded hygienic and infection guidelines for HMBs, which will serve as a basis for the implementation of HDMB in the context of the intervention. For this purpose, internationally available guidelines on the operation of HDMBs, national recommendations, and the scientific literature with a special focus on microbiological screening and pasteurisation will be reviewed. The manual will include guidelines for the requirements of milk donors and their recruitment, hygienic aspects of collection, transport, processing and storage, as well as recommendations for microbiological testing of DHM. The final manuscript will be evaluated and approved by national experts in this field from different professions, including clinically active neonatologists and infectiologists, as well as microbiologists and hygienists. Evaluation of the manual will be conducted via a Delphi technique. Comments and remarks of all experts will be collected in a first round. In a second round, the revised manuscript will be checked again and unresolved issues will then be listed and discussed. The authors will record these issues and show, in a last step, how they will solve these open issues in the manuscript. Finally, the manual and documentation of the Delphi technique will be agreed upon by all involved experts. Apart from this manual, a Hazard Analysis and Critical Control Point (HACCP) concept will be developed, which is required by law to ensure food safety. This is a systematic approach aiming to identify, evaluate, and control possible food safety hazards throughout all processes from collection to application of DHM.

### Intervention development

The intervention development will occur through an interdisciplinary and multi-professional team. Among them will be physicians, nurses with experience in neonatology, psychologists, sociologists and behavioural economists. Based on the surveys and accompanied by input from mothers of VLBW infants, additional NICU nurses, and physicians, the individual components of the intervention (Table 1) will be developed in a participatory manner. The study will be accompanied by parent representatives and a scientific advisory board consisting of the relevant scientific societies.

**Table 1:** Preliminary elements of the structured lactation and breastfeeding support programme.

Written standard operating procedure (SOP) for breastfeeding promotion. Interdisciplinary training and information for the professional groups involved in the care of newborns in the NICU (midwives, nurses and physicians) [32]. The aim of this training is to convey the physiological background of lactation and present the current state of studies on the benefits of providing MOM/HDM, as well as promoting the praxis of early initiation of lactation, maintenance of lactation and coping with problems. Prenatal education of mothers and family members by the neonatologist during the consultation (possible in approx. 50% of cases, as there is sufficient lead time before delivery) [29, 35]. Support of early and continuous skin-to-skin contact with the infant(s). Early initiation of the first milk expression and breastfeeding [24]. Exclusive administration of MOM/HDM, unless medically contra-indicated. Support for the mother through an app (pre- and post-natal) that provides answers to the most important questions about breastfeeding and solutions to breastfeeding-related challenges (in the form of informational video clips), motivates breastfeeding and later reminds the mother to pump, and facilitates documentation of the amount of milk.

### Ethics, public engagement and dissemination

A positive ethical vote for the study by the Ethics Committee of the Medical Faculty of the University of Cologne has been obtained (no: 20–1547) and for conducting the interviews with the mothers additionally at Bielefeld university (2020–147). Participation in all surveys will be voluntary. In all pseudonymous surveys, which only take place with written consent, there will be the possibility to withdraw one’s consent at any time without repercussions. All surveys will be data protection compliant and the storage, processing, and deletion of research data will be carried out in accordance with the current legal situation.

Public engagement and dissemination of the results will occur on different channels depending on the target group. As instruments, a homepage will be implemented, which will explicitly address affected parents as well as medical staff. Here, the course of the project as well as relevant results will be published in target group adapted language. In addition to the homepage, an Instagram account will be created to inform the public here as well and to offer a low-threshold opportunity to promote contact between science and the public. This instrument is intended to encourage the target group to play a significant role in shaping the development of the intervention and to make their opinions known, e.g., by voting or giving feedback. The results of the NICU head survey will be reflected back to all leaders in the form of a fact sheet in an anonymous and aggregated form. The scientific community will be informed about scientific publications and the presentation of results at specialist conferences.

## Supporting information

S1 File (DOCX)

S2 File (DOCX)

S3 File (DOCX)

S4 File (PDF)
