# Supplementary material for: Structured lactation support and human donor milk for German NICUs—Protocol on an intervention design based on a multidimensional status quo and needs assessment (Neo-MILK)
Source: PLoS One. 2023 Apr 27;18(4):e0284621. doi: 10.1371/journal.pone.0284621 (PMC10138472; doi:10.1371/journal.pone.0284621)
Supplement: S1 File — (DOCX) [file pone.0284621.s001.docx]

German Clinical Trials Register

https://drks.de/search/de/trial/DRKS00024799
