# Supplementary material for: Structured lactation support and human donor milk for German NICUs—Protocol on an intervention design based on a multidimensional status quo and needs assessment (Neo-MILK)
Source: PLoS One. 2023 Apr 27;18(4):e0284621. doi: 10.1371/journal.pone.0284621 (PMC10138472; doi:10.1371/journal.pone.0284621)
Supplement: S2 File — (DOCX) [file pone.0284621.s002.docx]

***Attachment 2***

**Checklist for study protocols for monocentre and multicentre prospective data collections
(based on the WHO-template)**

1. **Project title, version number, version date**

NEO-MILK - Breast milk banks: Implementation and promotion of lactation in German neonatal intensive care units

1. **Summary of the project (abstract or tabular synopsis)**

It is undisputed that breast milk is the best nutrition for newborns. Especially for premature infants, breast milk is essential for the prevention of vital infections (such as necrotising enterocolitis (NEC)), the modulation of the immune system and cognitive development. For more than 15 years, the WHO has been recommending the exclusive feeding of mother's milk. To this end, it is necessary to provide early lactation support to the mothers of premature babies. If it is not possible for the mother to supply her child with breast milk by herself, the supply of human donor milk should be preferred. In Germany, this is currently only possible in a few neonatal intensive care units (NICUs) due to structural, legal and financial obstacles. Furthermore, there is no structured care for mothers of preterm infants in Germany with regard to lactation. The aim of the new form of care: every premature baby in Germany has access to breast milk or human donor milk from the first day of life. This will lead to a short-term improvement in the medical outcome (e.g. avoidance of infections) of the premature baby and, in the long term, to the greatest possible avoidance of artificially produced food (formula) in German NICUs through structured lactation and breastfeeding support and the establishment of human donor milk banks through the creation of legal and structural foundations.

The work packages of phase 1 are described here and submitted for evaluation. The intervention and evaluation (phase 2) will be submitted for evaluation when the legal framework has been further defined and thus the intervention has been defined in detail.

*Object of investigation of phase 1:*

1: Survey of existing human donor milk banks in cooperation with the FMBI n = 30

2: Survey of mothers of premature babies under 1,500 grams via the cooperating health insurance funds (DAK, TK, AOK Rheinland and pronova BKK): n = 2,700

3: Survey of medical and nursing directors in German neonatal intensive care units (NICU): n = 211 NICUs

4. Conducting interviews with "experts" in the field of human donor milk banks: n = 6 (or until saturation) together with the University of Bielefeld

5. Interviews with mothers of premature babies on the topic of breast milk and breastfeeding: n = 12 (or until satiation) together with the University of Bielefeld

1. **Responsibilities**
   - Director of Studies

Dr. Nadine Scholten, Institute for Medical Sociology, Health Services Research and Rehabilitation Science (IMVR), University Hospital Cologne, University of Cologne (UzK)

- - participating scientists

| **Name** | **Institution** | **Telefon, Fax, E-Mail** | **Responsibility/Role** |
| --- | --- | --- | --- |
| Prof. Dr. Andreas Müller, Dr. Till Dresbach | University Hospital Bonn, Neonatology | Tel.: +49228 287 33408 Fax: +49228 287 33296  neonatologie@ukb.uni-bonn.de | WP 2: Development of the standard "Implementation of the human donor milk bank" and neonatological monitoring of the overall project.  WP 4: Monitoring of the implementation of the intervention |
| Prof. Dr. Nicole Ernstmann, Dr. Antje Hammer | Institute for Patient Safety Bonn | Tel.: +49228 28715763  nicole.ernstmann@ ukbonn.de  antje.hammer@ ukbonn.de  Tel.: +49228 28713980 | WP 2: Monitoring with a focus on patient safety |
| Prof. Dr. Friederike Eyssel | CITEC Center of Excellence Cognitive Interaction Technology, University Bielefeld | Tel.: +49521 106 12044  feyssel@cit-ec.uni-bielefeld.de | WP 3: Concept for breastfeeding promotion |
| Prof. Dr. Martin Hellmich | IMSB, Statistics, University of Cologne | Tel.: +49221 47833409 Fax: +49221 47833420  martin.hellmich@uni-koeln.de | WP 5: Methodological support for the monitoring of the evaluation and the overall project |
| Dr. Angela Kribs | University Hospital Cologne, Neonatology and Paediatric Intensive Care Unit | Tel.: +49 221 478 85663  angela.kribs@uk-koeln.de | Neonatological monitoring of the overall project from the nursing perspective |
| Prof. Dr. Juliane Köberlein-Neu | Bergisches Kompetenzzentrum Health Economics and Health Services Research, University of Wuppertal | Tel.: +49202 439 1381  Fax: (0202) 4391384  koeberlein@wiwi.uni-wuppertal.de | WP 5: Formative and summative Evaluation |
| Prof. Dr. Katharina Lugani | Medical Law, Heinrich Heine University Düsseldorf | Tel.: +49211 8111429 Fax: +49211 8111450  ls.hilbig-lugani@uni-duesseldorf.de | WP 2: Legal support for the establishment of the standard "Implementation of a human donor milk bank |
| Prof. Dr. Eva Mildenberger | Mainz University Medical Centre, Neonatology | Tel.: +49 6131 17 5890  eva.mildenberger@unimedizin-mainz.de | WP 3: Concept for breastfeeding promotion  WP 4: Monitoring implementation of the intervention |
| PD Dr. Jens Ulrich Rüffer, Katja Matthias | TAKEPART Media + Science GmbH | Tel.: +49221 2925760  rueffer@takepart- media.de | WP3: Development of app and film footage |
| Dr. Nadine Scholten | University Hospital Cologne, Institute for Medical Sociology, Health Services Research and Rehabilitation Science (IMVR) | Tel.: +49221 47897156 nadine.scholten@uk-koeln.de | Project management/  Consortium management (WP1-AP4)  WP 1: Status quo and implementation barriers  WP 4: Implementation of the intervention |
| Anne Sunder-Plaßmann, M. A. | Hamburg | Tel.: +49 (0)40 38631459  a.sunder-plassmann@gmx.net | WP 2: Accompanying development of a standard for the implementation of human donor milk banks |
| Prof. Dr. Daniel Wiesen | Behavioral Management Science und C-SEB, University of Köln | Tel.: +49221 470 89171  Fax +49 221 470 89259  wiesen@wiso.uni-koeln.de | WP 2 and WP 3: Accompanying behavioural change |
| Christoph Rupprecht | AOK Rheinland/Hamburg | Tel.: 0211-8791-1154  Fax: 0211-8791-1145  christoph.rupprecht@rh.aok.de | WP1: Status quo and implementation hurdles |
| Dr. Melanie Klein | DAK | Wissenschaftlicher Beirat: beirat@dak.de | WP1: Status quo and implementation hurdles |
| Dr. Dirk-Horenkamp-Sonntag | Techniker Krankenkasse | Tel.: 040 6909-2812  dr.dirk.horenkamp-  sonntag@tk.de | WP1: Status quo and implementation hurdles |

- - Facilities involved (e.g. laboratory, imaging)

does not apply

- - Financing

Innovationsfonds FKZ: 01NVF19027

All study components are financed by the public sponsor.

- - Registration in a publicly accessible study register

Registration in the German Clinical Trials Register: to follow

1. **Scientific background**

- State of research (with literature references) and derivation of the research question (rationale)

It is undisputed that breast milk is the best nutrition for every newborn. The WHO has been recommending exclusive feeding with breast milk from the first day of life for over 15 years [1]. For the further development of vulnerable newborns, such as children with a birth weight of less than 1,500 grams or newborns with congenital diseases, optimal nutrition with breast milk is particularly decisive. In Germany, these newborns are cared for in neonatal intensive care units (NICUs) after birth. Currently, there is no structured care for mothers of preterm infants in Germany with regard to lactation. To stimulate milk let-down, mothers need to be encouraged to pump milk immediately after birth and to continue to do so regularly. Early initiation immediately after birth has been shown to increase the amount of breast milk and promote an early supply of only breast milk [2]. This structured promotion of lactation is particularly necessary for mothers of premature infants, as the production of breast milk may be delayed and impeded [2]. Ideally, in the absence of breast milk, the preterm infant is first supplied with human donor milk after birth, so that it can subsequently be supplied with breast milk as soon as possible. For this purpose, it is necessary to support the mothers in lactation at an early stage [3]. If the child is initially fed with artificially prepared food (formula), the probability increases that it will not be fed exclusively with breast milk at the time of discharge [3]. If breastfeeding readiness and lactation are supported in a structured way, the majority of children in NICUs can be fed directly or after a short time with their own mother's breast milk. Internationally, evidence-based concepts for promoting lactation and breastfeeding readiness in NICUs are already available [4]. If it is fundamentally or at least initially not possible for the mother to feed her child with her own breast milk, feeding with human donor milk should be preferred. In Germany, this is currently only possible in a few NICUs due to structural, legal and financial obstacles. Of the current 211 Level I and Level II NICUs in Germany, 22 have a women's milk bank and are organised in the Women's Milk Bank Initiative (FMBI) [5]. Due to the lack of standards for the implementation/establishment of a human donor milk bank in Germany, these are very differently designed, very unevenly distributed regionally (15 of 22 are located in the new federal states) and primarily at very large (university) hospitals [5]. The currently existing donor milk banks are based on the initiatives of committed neonatologists. Internationally, on the other hand, human donor milk banks have already been established in some areas and evidence-based concepts for the implementation of human donor milk banks are available (e.g. USA, Switzerland, Austria or Great Britain) [6-8]. The most recent guideline for Germany dates from 1998 [11]. An adaptation of this guideline to the current scientific findings and circumstances therefore seems urgently necessary.

In the preparation of the standard for the implementation and operation of a human donor milk bank, the expertise of the already existing breast milk banks is to be drawn upon via the Women's Milk Bank Initiative FMBI. For this purpose, they will be asked in writing and interviews will be conducted with selected experts (4). To identify possible implementation barriers, all medical and nursing directors of level 1 and level 2 NICUs in Germany will be interviewed (3).

The creation of the structured breastfeeding promotion programme is also based on the wishes, attitudes and experiences of the women concerned. These are to be collected on the one hand through structured interviews (5) and on the other hand through a written survey via the cooperating health insurance fund (2).

1. **Project goals**

- Primary/secondary goals

The primary aim of the qualitative and quantitative surveys is to gain scientific knowledge on the conception of the standard for the implementation of donor milk banks and on structured breastfeeding promotion.

1. **Targets**

- Primary/secondary goals

Due to the explorative and partly qualitative approach, these cannot be determined in advance.

1. **Study population**
   - Inclusion and exclusion criteria

1. Person responsible for the human donor milk banks (key person survey) and member of the FMBI, existence of a declaration of consent

2. Member of one of the cooperating health insurance funds (DAK, TK, AOK Rheinland and pronova BKK), mothers of premature babies with a birth weight of less than 1,500 grams, whose child is between six and 18 months old at the time of the survey. The following ICD-10 codes are used for identification: P07.01, P07.02, P07.10 and P07.11, anonymous interview with implicit declaration of consent.

3. Survey of medical and nursing directors in German neonatal intensive care units: n = 211 NICUs, anonymous survey with implicit informed consent

4. Interviews with "experts" in the field of human donor milk banks and availability of a valid consent form

WP5. Mothers of premature babies under 1,500 grams, age of the child under 18 months, presence of a valid PA, sufficient language skills.

- - Number of study participants and recruitment measures

1. Survey of existing human donor milk banks in cooperation with the FMBI: n = 30

2. Survey of mothers of premature babies under 1,500 grams via the cooperating health insurance funds (DAK, TK, AOK Rheinland and pronova BKK): n = 2,700

3. Survey of medical and nursing staff in German neonatal intensive care units: n = 211 NICUs, full survey

4. Conduct interviews with experts in the field of human donor milk banking. Recruitment via written survey (1)

5. Conducting interviews with mothers of preterm infants on the topic of breast milk and styles: n = 12, recruitment via social media and the cooperation partners

1. **Methodology and implementation**
   - monocentric/multicentric

multicentric

- - Procedure for informing and obtaining consent

The anonymous surveys are conducted without asking for consent. It is explained in the study information that implicit consent is assumed when the questionnaire is returned (2 and 3).

The pseudonymous data collection will only take place if a valid consent form is available (qualitative interviews and survey of the existing donor milk banks 4 and 5). With the consent form, the participant is informed in writing on the basis of the study information. Participation is voluntary and consent can be withdrawn at any time.

- - Description of data sources (medical records, questionnaires, etc.)

1. Survey of existing human donor milk banks: quantitative data collection using a standardised questionnaire.

2. Survey of mothers of premature babies under 1,500 grams via the cooperating health insurance funds (DAK, TK, AOK Rheinland and pronova BKK): quantitative data collection using a standardised questionnaire.

3. Survey of medical and nursing staff in German neonatal intensive care units: quantitative data collection using a standardised questionnaire.

4. Interviewing "experts" in the field of donor milk banks: qualitative interview data, audio files, transcripts.

5. Conducting interviews with mothers of premature babies on the topic of breast milk and style-len: qualitative interview data, audio files, transcripts

- - List/description of the data to be collected

The finalised questionnaires and interview guidelines will be made available to the Ethics Committee.

- - If applicable: time schedule (appointments) for the individual study participant (Flow chart)

Does not apply


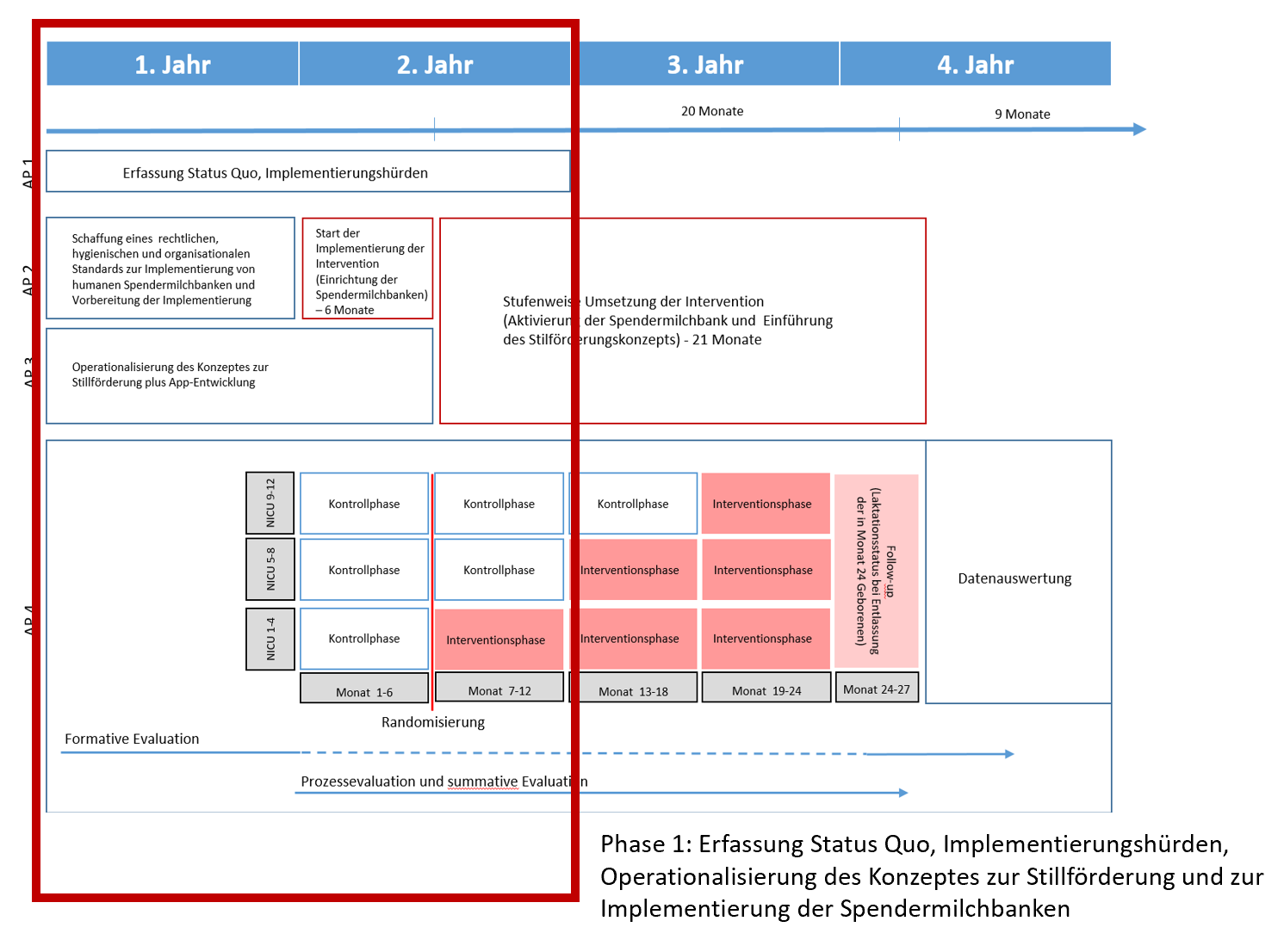


- - Expected end of data collection

End of phase 1: 31. 12.2023

1. **Benefit-risk assessment**
   - Individual benefit associated with study participation

Participation in the survey of mothers via the cooperating health insurance companies is rewarded with a 10 euro voucher (DM/Amazon).

An expense allowance of 75 euros is provided for the participation in the mothers' interviews.

An expense allowance of 150 euros is provided for the expert interviews.

- - Burdens and risks associated with study participation

Participation in all surveys is voluntary. Apart from the time involved, no further burden is expected for the study participant. The questionnaires / interview guidelines will be made available to the Ethics Committee before the survey/interviews are conducted.

- - Statement on medical justifiability

Not applicable.

1. **Biometry**
   - exploratory study: explanation of the statistical methodology:

1. Survey of existing human donor milk banks in cooperation with the FMBI: n = 30

2. Survey of mothers of premature babies under 1,500 grams via the cooperating health insurance funds (DAK, TK, AOK Rheinland and pronova BKK): n = 2,700

3. Survey of medical and nursing directors in German neonatal intensive care units: n = 211 NICUs

4. Conducting interviews with "experts" in the field of human donor milk banks: n = 6 (or until saturation).

5. Conducting interviews with mothers of preterm infants on the topic of breast milk and style: n = 12

The surveys 1) 2) and 3) are full surveys. These are necessary in order to generate sufficient data material to be able to calculate regional multivariate models, for example, in view of the expected response.

With regard to the sample size of qualitative research, in contrast to quantitative research, there are no concrete indications. The sample is often significantly smaller than in quantitative research. This can be justified on the one hand by the available resources and on the other hand by content-related considerations. The evaluation of non-standardised data is usually much more time-consuming. However, a large sample is also not necessary from a methodological point of view, since a profound spectrum of information about the subject area can already be achieved with a few test persons (goal of theoretical generalisation of the sample results). Based on the assumption of theoretical saturation, only redundant information would result from more interviews. Overall, no concrete guidelines can be given for determining the sample size in qualitative research. However, there are recommendations for research practice. First of all, the available resources must be considered, since conducting qualitative interviews is very time-consuming and cost-intensive. Furthermore, the scope of the research question is important. The so-called theoretical saturation occurs sooner, the more specific the question is [1].

Depending on the evaluation method, the research literature suggests conducting between 5 and 50 interviews [2]. With regard to the evaluation of interviews with the help of qualitative content analysis, a study by Guest et al. showed that most of the assigned category codes were already covered after 6 interviews [3]. This illustrates that the important topics of the object of study can be covered even with very small sample sizes [1]. The number of cases chosen for the subjects in this study can therefore be justified according to the current state of science.

1. Akremi L: Stichprobenziehung in der qualitativen Sozialforschung. In: Baur N, Blasius J (eds.): Handbuch der Methoden der empirischen Sozialforschung. Wiesbaden: Springer Fachmedien 2014; 265–282.

2. Mason M: Sample Size and Saturation in PhD Studies Using Qualitative Interviews. Forum: Qualitative Sozialforschung 2010; 11(3).

3. Guest G, Bunce A, Johnson L: How Many Interviews Are Enough? Field Methods 2006; 18(1): 59–82.

1. **Data management and data protection**

- responsible for data processing; data protection officer of Initiator and Study Centre^[[1]](#footnote-1)^

Head of study Dr. Nadine Scholten, data trustee: Markus Alich (Care Research Lab)

1.: Survey of existing donor milk banks: Pseudonymous survey with obtaining a declaration of consent (EV). The pseudonymisation list is administered in the IMVR by an employee of the Care Research Lab who is not involved in the project (data trustee: Markus Alich). The project staff have no access to it. Before the data analysis by the researchers of the project, a second pseudonymisation is carried out by the data trustee.

Rights to information (including the right to a copy of the data free of charge), correction, deletion and restriction of data processing in accordance with the DSGVO are granted. The declaration of consent can be revoked at any time, which leads to the deletion of the data.

With the EV, the willingness to participate in an interview is requested at the same time.

2.: Survey of mothers of premature babies: The survey takes place anonymously, whereby the survey documents prepared by the IMVR are sent by the participating health insurance funds to mothers of premature babies with a birth weight of less than 1,500 grams. The IMVR does not receive any information from the health insurer that directly identifies the person. The mothers send the anonymous questionnaire back to the IMVR, whereby care is taken when preparing the questionnaires not to collect any information that directly identifies a person. It is pointed out in the free texts that no names should be mentioned. In order to send the voucher to the participating mothers, a form for the transmission of the e-mail address is enclosed. This should be enclosed in a sealed envelope with the questionnaire. The envelope is opened at the trust centre and processed separately from the questionnaire. There is no link between the questionnaire and the e-mail address. The e-mail address will only be used to transmit the voucher code.

3. Survey of medical and nursing staff in German neonatal intensive care units: The survey is anonymous and includes a response control.

When preparing the questionnaires, care is taken to ensure that no information is collected that directly identifies a person. It is pointed out in the free texts that no names should be mentioned. The survey will be conducted in accordance with the Total Design Method according to Dillman, with up to four postal survey waves. Recruitment will take place via publicly available contact data. The following procedure is planned: The medical and nursing staff will receive the letter and the questionnaire and will be informed that their participation in the survey will be interpreted as an implicit declaration of consent to the procedure according to the study information and that the survey itself will therefore take place anonymously. The stamped envelopes of the leaders are numbered on the outside so that the IMVR can check the return of the questionnaire. This is done as follows: 1.) The numbers of the envelopes received in the IMVR are deleted from the original address list 2.) Reminders and thus the repeated mailing of questionnaires are thus only based on the remaining addresses. 3) After completion of the (up to) four survey waves, the address list will be deleted and only then will the envelopes be opened and the anonymous questionnaires evaluated. 4) All incoming envelopes will be deleted until then. All incoming envelopes are kept in a locked storage room at the IMVR until then. With the help of this procedure, targeted reminders are possible and at the same time it is avoided that those leaders who have already participated are written to several times and possibly harassed. The primary goal of the chosen procedure is to achieve the highest possible participation in the survey while maintaining anonymity. A procedure without response control and targeted reminder waves jeopardises the response rate to be achieved.

4. Conducting interviews with experts in the field of human donor milk banks and 5. conducting interviews with mothers of premature babies on the topic of breast milk and breastfeeding: For evaluation purposes, audio recordings will be made during the interviews, after the participants have been informed and have given their consent. The recordings are pseudonymised and handed over to the external service provider for transcription. For this purpose, a contract will be concluded with the service provider to ensure compliance with data protection regulations. Only the anonymous transcripts are used for the evaluations.

Rights to information (including the right to a free copy of the data), correction, deletion and restriction of data processing in accordance with the GDPR are granted. The declaration of consent can be revoked at any time, which leads to the deletion of the data.

1. **Signatures: Director of Studies (applicant)**

**Attachment: List of participating study centres (for multi-centre studies)**

**University Bielefeld:**

| Prof. Dr. Friederike Eyssel | CITEC Center of Excellence Cognitive Interaction Technology, Universität Bielefeld | Tel.: +49521 106 12044  feyssel@cit-ec.uni-bielefeld.de | WP 3: Concept for breastfeeding promotion |
| --- | --- | --- | --- |

1. The data controller is at least also the initiator (sponsor or other carrier), because the initiator decides - if necessary together with others - on the purposes and means of data processing (cf. the definition in Art. 4 No. 7 DSGVO). Therefore, the initiator and its data protection officer must be named first. In addition, the responsibility of the local study centre can also be considered.

   It is always advisable to name a contact person in the study centre for exercising the rights according to the DSGVO (information, deletion, etc.) so that the initiator, who does not have access to the personal data, does not necessarily have to be contacted. [↑](#footnote-ref-1)
