# Supplementary material for: Structured lactation support and human donor milk for German NICUs—Protocol on an intervention design based on a multidimensional status quo and needs assessment (Neo-MILK)
Source: PLoS One. 2023 Apr 27;18(4):e0284621. doi: 10.1371/journal.pone.0284621 (PMC10138472; doi:10.1371/journal.pone.0284621)
Supplement: S3 File — (DOCX) [file pone.0284621.s003.docx]

**GUIDANCE FOR CLINICAL TRIAL PROTOCOLS**

**SPIRIT (Standard Protocol Items: Recommendations for Interventional Trials)**

The [SPIRIT 2013 Statement](https://www.spirit-statement.org/publications-downloads/)provides evidence-based recommendations for the minimum content of a clinical trial protocol. SPIRIT is [widely endorsed](https://www.spirit-statement.org/about-spirit/spirit-endorsement/) as an international standard for trial protocols.

| NO clinical trial that can be described using the SPIRIT checklist was submitted here. Rather, it is a participatory intervention development based on different status quo surveys and theoretical work packages. This intervention will then be evaluated as an intervention in a cluster randomized controlled study in a second step. An intervention protocol will be submitted in due course. |
| --- |
