# Supplementary material for: Structured lactation support and human donor milk for German NICUs—Protocol on an intervention design based on a multidimensional status quo and needs assessment (Neo-MILK)
Source: PLoS One. 2023 Apr 27;18(4):e0284621. doi: 10.1371/journal.pone.0284621 (PMC10138472; doi:10.1371/journal.pone.0284621)
Supplement: S4 File — (PDF) [file pone.0284621.s004.pdf]

Anlage 2

Checkliste für Studienprotokolle für mono- und multizentrische *prospektive Datenerhebungen*
(in Anlehnung an das WHO-Template)

1. Projekttitel, Versionsnummer, Versionsdatum

NEO-MILK – Muttermilchbanken: Implementierung und Förderung der Laktation auf deutschen neonatologischen Intensivstationen

2. Zusammenfassung des Projekts (Abstract oder tabellarische Synopse)

Unbestritten ist die Muttermilch die beste Ernährung von Neugeborenen. Gerade für frühgeborene Kinder ist die Muttermilch essentiell für die Verhinderung vital bedrohlicher Infektionen (wie z. B. die nekrotisierende Enterokolitis (NEC)), die Modulation des Immunsystems und die kognitive Entwicklung. Die WHO empfiehlt bereits seit über 15 Jahren die ausschließliche Ernährung mit Muttermilch. Hierzu ist es notwendig, den Müttern von Frühgeborenen frühzeitig Unterstützung zur Laktation zukommen zu lassen. Ist es der Mutter nicht möglich, ihr Kind selbst mit Muttermilch zu versorgen, ist die Versorgung mit humaner Spendermilch zu präferieren. Dies ist in Deutschland aktuell aufgrund struktureller, rechtlicher und finanzieller Hemmnisse nur in wenigen neonatologischen Intensivstationen (NICUs) möglich. Des Weiteren findet in Deutschland keine strukturierte Betreuung der Mütter von Frühgeborenen im Hinblick auf die Laktation statt. Ziel der neuen Versorgungsform: Jedes Frühgeborene in Deutschland erhält ab dem ersten Lebenstag Zugang zu Muttermilch oder humaner Spendermilch. Hierdurch kommt es kurzfristig zur Verbesserung des medizinischen Outcomes (z. B. Vermeidung von Infektionen) des Frühgeborenen und langfristig zu einer weitestgehenden Vermeidung von künstlich hergestellter Nahrung (Formula) auf deutschen NICUs durch die strukturierte Laktations- und Stillförderung und die Etablierung von humanen Spendermilchbanken über die Schaffung von rechtlichen und strukturellen Grundlagen.

Hier beschrieben und zur Bewertung vorgelegt werden die Arbeitspakete der Phase 1. Die Intervention und Evaluation (Phase 2) wird zur Bewertung vorgelegt, wenn der rechtliche Rahmen näher definiert und dadurch die Intervention im Detail definiert wurde.

Untersuchungsgegenstand der Phase 1:

1: Befragung der vorhandenen humanen Spendermilchbanken in Kooperation mit der FMBI n = 30

2: Befragung der Mütter von Frühgeborenen unter 1.500 Gramm über die kooperierenden Krankenkassen (DAK, TK, AOK Rheinland und pronova BKK): n = 2700

3: Befragung der ärztlichen und pflegerischen Leitungen auf deutschen neonatologischen Intensivstationen (NICU): n = 211 NICUs

4. Durchführung von Interviews mit „Experten“ im Bereich humane Spendermilchbanken: n = 6 (bzw. bis zur Sättigung) gemeinsam mit der Uni Bielefeld

5. Interviews mit Müttern von Frühgeborenen zum Thema Muttermilch und Stillen: n = 12 (bzw. bis zur Sättigung) gemeinsam mit der Uni Bielefeld

3. Verantwortlichkeiten

- Studienleiter/in

Dr. Nadine Scholten, Institut für Medizinsoziologie, Versorgungsforschung und Rehabilitationswissenschaft (IMVR), Universitätsklinik Köln, Universität zu Köln (UzK)

- beteiligte Wissenschaftler/innen

**Name**	**Institution**	**Telefon, Fax, E-Mail**	**Verantwortlichkeit/Rolle**
Prof. Dr. Andreas Müller, Dr. Till Dresbach	Universitätsklinikum Bonn, Neonatologie	Tel.: +49228 287 33408<br>Fax: +49228 287 33296<br>neonatologie@ukb.uni-bonn.de	AP 2: Entwicklung des Standards „Implementierung humane Spendermilchbank“ und neonatologische Begleitung des Gesamtprojektes<br>AP 4: Begleitung der Implementierung der Intervention
Prof. Dr. Nicole Ernstmann, Dr. Antje Hammer	Institut für Patientensicherheit Bonn	Tel.: +49228 28715763<br>nicole.ernstmann@ukbonn.de<br>antje.hammer@ukbonn.de<br>Tel.: +49228 28713980	AP 2: Begleitung mit Schwerpunkt auf Patientensicherheit
Prof. Dr. Friederike Eyssel	CITEC Center of Excellence Cognitive Interaction Technology, Universität Bielefeld	Tel.: +49521 106 12044<br>feyssel@cit-ec.uni-bielefeld.de	AP 3: Konzept zur Stillförderung
Prof. Dr. Martin Hellmich	IMSB, Statistik, Universität zu Köln	Tel.: +49221 47833409<br>Fax: +49221 47833420<br>martin.hellmich@uni-koeln.de	AP 5: Methodische Begleitung der Begleitung der Evaluation und des Gesamtprojektes
Dr. Angela Kribs	Universitätsklinik Köln, Neonatologie und pädiatrische Intensivstation	Tel.: +49 221 478 85663<br>angela.kribs@uk-koeln.de	Neonatologische Begleitung des Gesamtprojektes aus Sicht der Pflege
Prof. Dr. Juliane Köberlein-Neu	Bergisches Kompetenzzentrum für Gesundheitsökonomik und Versorgungs-forschung, Bergische Universität Wuppertal	Tel.: +49202 439 1381<br>Fax: (0202) 4391384<br>koeberlein@wiwi.uni-wuppertal.de	AP 5: Formative und summative Evaluation
Prof. Dr. Katharina Lugani	Medizinrecht, Heinrich Heine Universität Düsseldorf	Tel.: +49211 8111429<br>Fax: +49211 8111450<br>ls.hilbig-lugani@uni-duesseldorf.de	AP 2: Rechtliche Begleitung der Festlegung des Standards „Implementierung humane Spendermilchbank“
Prof. Dr. Eva Mildenberger	Universitätsmedizin Mainz, Neonatologie	Tel.: +49 6131 17 5890<br>eva.mildenberger@unimedizin-mainz.de	AP 3: Konzept zur Stillförderung<br>AP 4: Begleitung Implementierung der Intervention
PD Dr. Jens Ulrich Rüffer, Katja Matthias	TAKEPART Media + Science GmbH	Tel.: +49221 2925760<br>rueffer@takepart-media.de	AP3: Entwicklung App und Filmmaterial

Dr. Nadine Scholten	Universitätsklinik Köln, Institut für Medizinsoziologie, Versorgungsforschung und Rehabilitations-wissenschaft (IMVR)	Tel.: +49221 47897156 nadine.scholten@uk-koeln.de	Projektleitung/ Konsortialführung (AP1-AP4) AP 1: Status Quo und Implementierungshürden AP 4: Implementierung der Intervention
Anne Sunder-Plaßmann, M. A.	Hamburg	Tel.: +49 (0)40 38631459 a.sunder-plassmann@gmx.net	AP 2: Begleitung Entwicklung eines Standards zur Implementierung von humanen Spendermilchbanken
Prof. Dr. Daniel Wiesen	Behavioral Management Science und C-SEB, Universität zu Köln	Tel.: +49221 470 89171 Fax +49 221 470 89259 wiesen@wiso.uni-koeln.de	AP 2 und AP 3: Begleitung Verhaltensänderung
Christoph Rupprecht	AOK Rheinland/Hamburg	Tel.: 0211-8791-1154 Fax: 0211-8791-1145 christoph.rupprecht@rh.aok.de	AP1: Status Quo und Implementierungshürden
Dr. Melanie Klein	DAK	Wissenschaftlicher Beirat: beirat@dak.de	AP1: Status Quo und Implementierungshürden
Dr. Dirk-Horenkamp-Sonntag	Techniker Krankenkasse	Tel.: 040 6909-2812 dr.dirk.horenkamp-sonntag@tk.de	AP1: Status Quo und Implementierungshürden

- beteiligte Einrichtungen (z.B. Labor, Bildgebung)

trifft nicht zu

- Finanzierung

Innovationsfonds FKZ: 01NVF19027

Alle Studienbestandteile werden über den öffentlichen Förderer finanziert.

- Registrierung in einem öffentlich zugänglichen Studienregister

Registrierung im deutschen Register klinischer Studien: folgt

4. Wissenschaftlicher Hintergrund

- Stand der Forschung (mit Literaturangaben) und Ableitung der Fragestellung (Rationale)

Unbestritten ist Muttermilch die beste Ernährung für jedes Neugeborene. Die WHO empfiehlt bereits seit über 15 Jahren die ausschließliche Ernährung mit Muttermilch ab dem ersten Lebenstag [1]. Für die weitere Entwicklung besonders entscheidend ist die optimale Ernährung mit Muttermilch für vulnerable Neugeborene, wie Kinder mit einem Geburtsgewicht unter 1.500 Gramm oder auch Neugeborene mit angeborenen Erkrankungen. Diese Neugeborenen werden in Deutschland nach der Geburt auf neonatologischen Intensivstationen (NICUs) versorgt. Aktuell findet in Deutschland keine strukturierte Betreuung der Mütter von Frühgeborenen im Hinblick auf die Laktation statt. Um den Milcheinschuss anzuregen, ist es notwendig, dass die Mütter direkt nach der Geburt dazu angehalten werden Milch abzupumpen und dies regelmäßig fortzuführen. Durch die frühzeitige Initiierung direkt nach Geburt kann nachweislich die Muttermilchmenge gesteigert und die frühzeitige Versorgung ausschließlich mit Muttermilch gefördert werden [2]. Diese strukturierte Förderung der Laktation ist besonders

bei Müttern von Frühgeborenen notwendig, da es hier vermehrt zu einer verzögerten und erschwerten Produktion von Muttermilch [2]. Im Idealfall wird das Frühgeborene im Falle eines Nichtvorhandenseins von Muttermilch zunächst nach der Geburt mit humaner Spendermilch versorgt, um nachfolgend zeitnah mit Muttermilch versorgt zu werden. Hierzu ist es notwendig, die Mütter frühzeitig bei der Laktation zu unterstützen [3]. Wird das Kind initial mit künstlich hergestellter Nahrung (Formula) ernährt, steigt die Wahrscheinlichkeit, dass es auch zum Zeitpunkt der Entlassung nicht ausschließlich mit Muttermilch ernährt wird [3]. Wird die Stillbereitschaft und Laktation strukturiert unterstützt, kann ein Großteil der Kinder auf NICUs direkt oder nach kurzer Zeit mit der Muttermilch der eigenen Mutter ernährt werden. International liegen bereits evidenzbasierte Konzepte zur Förderung der Laktation und Stillbereitschaft auf NICUs vor [4]. Ist es der Mutter grundsätzlich oder zumindest anfangs nicht möglich, ihr Kind mit eigener Muttermilch zu versorgen, ist die Ernährung mit humaner Spendermilch zu präferieren. Dies ist in Deutschland aktuell aufgrund struktureller, rechtlicher und finanzieller Hemmnisse nur in wenigen NICUs möglich. Von den aktuell 211 Level I und Level II NICUs in Deutschland haben 22 eine Frauenmilchbank und sind in der Frauenmilchbank-Initiative (FMBI) [5] organisiert. Aufgrund des bisher fehlenden Standards zur Implementierung/ zum Aufbau einer humanen Spendermilchbank in Deutschland sind diese sehr unterschiedlich ausgestaltet, regional sehr ungleich verteilt (15 von 22 liegen in den neuen Bundesländern) und vorrangig an sehr großen (Universitäts)Kliniken [5]. Die aktuell vorhandenen Spendermilchbanken beruhen auf den Initiativen von engagierten Neonatologinnen und Neonatologen. International sind dagegen humane Spendermilchbanken teilweise bereits flächendeckend etabliert und evidenzbasierte Konzepte zur Implementierung von humanen Spendermilchbanken liegen vor (z. B. USA, Schweiz, Österreich oder Großbritannien) [6–8]. Die für Deutschland aktuellste Leitlinie stammt aus dem Jahr 1998 stammt [11]. Eine Anpassung dieser an die aktuellen wissenschaftlichen Erkenntnisse und Gegebenheiten erscheint daher dringend notwendig.

In die Erstellung des Standards zur Implementierung bzw. zum Betrieb einer humanen Spendermilchbank, soll auf die Expertise der bereits vorhandenen Muttermilchbanken über die Frauenmilchbankinitiative FMBI zurückgegriffen werden. Hierzu sollen diese schriftlich befragt und in der Folge mit ausgewählten Experten Interviews durchgeführt werden (4). Zur Identifikation möglicher Implementierungshürden werden alle ärztlichen und pflegerischen Leitungen der Level 1 und Level 2 NICUs in Deutschland befragt werden (3)

Die Erstellung des strukturierten Stillförderungsprogamms basiert auch auf den Wünschen, Einstellungen und Erfahrungen betroffener Frauen. Diese sollen zum einen durch strukturierte Interviews (5) und zum anderen durch eine schriftliche Befragung über die kooperierenden Krankenkasse erhoben werden (2).

5. Projektziele

- primäre/sekundäre Ziele

Primäres Ziel der qualitativen, wie auch quantitativen Befragungen ist der wissenschaftliche Erkenntnisgewinn zur Konzeption des Standards zur Implementierung der Spendermilchbanken, wie auch zur strukturieren Stillförderung.

6. Zielgrößen

- primäre/sekundäre Zielgrößen

Aufgrund des explorativen und teilweise qualitativen Ansatzes können diese vorab nicht bestimmt werden.

7. Studienpopulation

- Ein- und Ausschlusskriterien

1. Verantwortlicher für die humane Spendermilchbanken (Schlüsselpersonenbefragung) und Mitglied im FMBI, Vorliegen einer EV

2. Mitglied in einer der kooperierenden Krankenkassen (DAK, TK, AOK Rheinland und pronova BKK), Mütter von Frühgeborenen mit einem Geburtsgewicht unter 1.500 Gramm, deren Kind zum Zeitpunkt der Befragung zwischen sechs und 18 Monaten alt ist. Die Identifizierung erfolgt über die folgenden ICD-10-Codes: P07.01, P07.02, P07.10 und P07.11, anonyme Befragung mit impliziter EV

3. Befragung der ärztlichen und pflegerischen Leitungen auf deutschen neonatologischen Intensivstationen: n = 211 NICUs, anonyme Befragung mit impliziter EV

4. Interviews mit „Experten" im Bereich humane Spendermilchbanken und Vorliegen einer gültigen EV

AP5. Mütter von Frühgeborenen unter 1.500 Gramm, Alter des Kindes unter 18 Monaten, Vorliegen einer gültigen EV, ausreichende Sprachkenntnisse

- Anzahl der Studienteilnehmer und Rekrutierungsmaßnahmen

1. Befragung der vorhandenen humanen Spendermilchbanken in Kooperation mit dem FMBI: n = 30

2.Befragung der Mütter von Frühgeborenen unter 1500 Gramm über die kooperierenden Krankenkassen (DAK, TK, AOK Rheinland und pronova BKK): n = 2700

3. Befragung der ärztlichen und pflegerischen Leitungen auf deutschen neonatologischen Intensivstationen: n = 211 NICUs, Vollerhebung

4. Durchführung Interviews mit Expertinnen und Experten im Bereich humane Spendermilchbank. Rekrutierung über die schriftliche Befragung (1)

5. Durchführung von Interviews mit Müttern von Frühgeborenen zum Thema Muttermilch und Stillen: n = 12, Rekrutierung über Social Media und die Kooperationspartner

8. Methodik und Durchführung

- monozentrisch/multizentrisch

multizentrisch

- Verfahren zur Aufklärung und Einholung der Einwilligung

Die anonymen Befragungen werden ohne Erhebung des Einverständnisses durchgeführt. Über die Studieninformation wird aufgeklärt, dass bei Zurücksenden des Fragebogens von einem impliziten Einverständnis ausgegangen wird (2 und 3)

Die pseudonymen Datenerhebungen finden nur bei Vorliegen einer gültigen Einverständniserklärung statt (qualitative Interviews sowie Befragung der bestehenden Spendermilchbanken 4 und5). Mit der

Einverständniserklärung wird der Teilnehmer schriftlich anhand der Studieninformation aufgeklärt. Die Teilnahme ist freiwillig und das Einverständnis kann jederzeit widerrufen werden.

- Beschreibung der Datenquellen (Krankenakten, Fragebögen etc.)

1.
Befragung der vorhandenen humanen Spendermilchbanken: quantitative Datenerhebung anhand eines standardisierten Fragebogens.
2. Befragung der Mütter von Frühgeborenen unter 1500 Gramm über die kooperierenden Krankenkassen (DAK, TK, AOK Rheinland und pronova BKK): quantitative Datenerhebung anhand eines standardisierten Fragebogens.
3. Befragung der ärztlichen und pflegerischen Leitungen auf deutschen neonatologischen Intensivstationen: quantitative Datenerhebung anhand eines standardisierten Fragebogens.
4.Befragung von „Experten“ im Bereich Spendermilchbanken: qualitative Interviewdaten, Audiodateien, Transkripte
5.Durchführung Interviews mit Müttern von Frühgeborenen zum Thema Muttermilch und Stillen: qualitative Interviewdaten, Audiodateien, Transkripte

- Auflistung/Beschreibung der zu erfassenden Daten

Die finalisierten Fragebögen bzw. Interviewleitfäden werden der Ethikkommission noch zur Verfügung gestellt werden

- Falls zutreffend: zeitlicher Ablauf (Termine) für den einzelnen Studienteilnehmer (Flow chart)

Trifft nicht zu

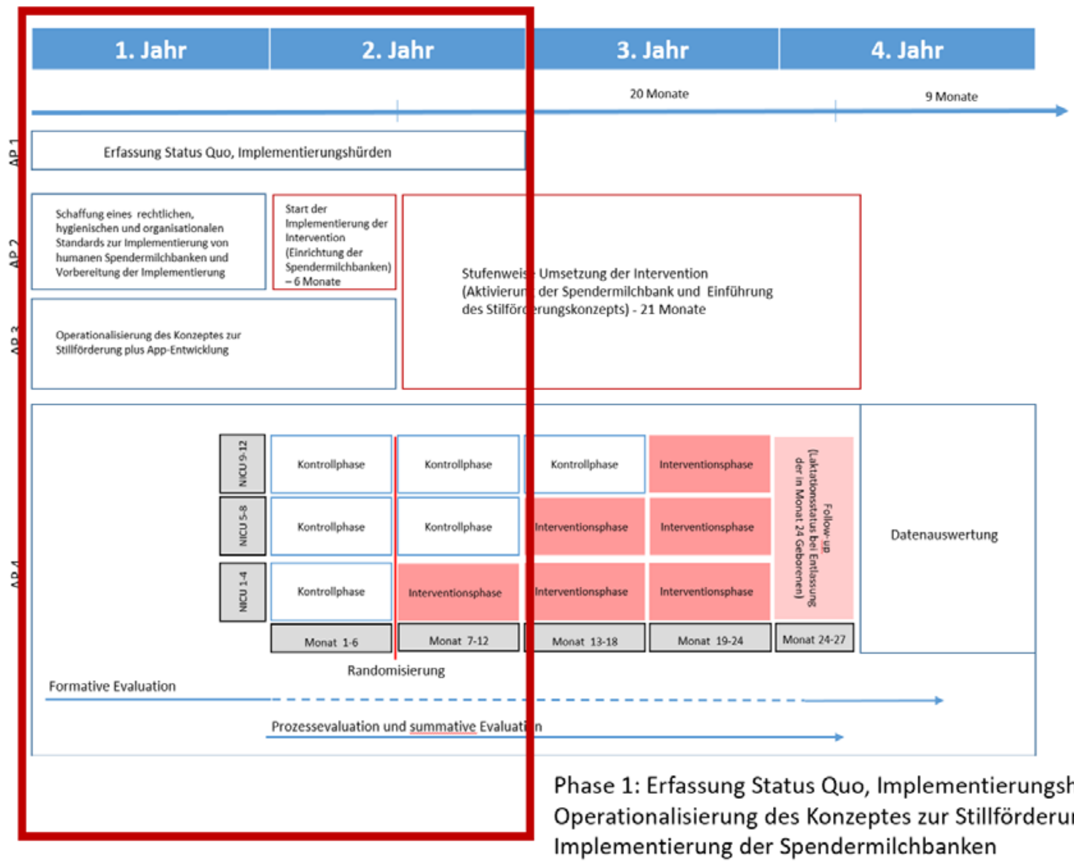

Phase 1: Erfassung Status Quo, Implementierungshürden, Operationalisierung des Konzeptes zur Stillförderung und zur Implementierung der Spendermilchbanken

- Voraussichtliches Ende der Datenerfassung

Beendigung Phase 1: 31. 12.2023

9. Nutzen-Risiko-Abwägung

- mit der Studienteilnahme verbundener individueller Nutzen

Die Teilnahme an der Befragung der Mütter über die kooperierenden Kassen wird mit einem 10 Euro Gutschein (DM/Amazon) honoriert.

Für die Teilnahme an den Interviews der Mütter ist eine Aufwandsentschädigung in Höhe von 75 Euro vorgesehen.

Für die Experteninterviews ist eine Aufwandsentschädigung in Höhe von 150 Euro vorgesehen.

- mit der Studienteilnahme verbundene Belastungen und Risiken

Die Teilnahme an allen Befragungen ist freiwillig. Bis auf den zeitlichen Aufwand ist keine weitere Belastung für den Studienteilnehmer zu erwarten. Die Fragebogen / Interviewleitfäden werden der Ethikkommission vor Durchführung der Befragung/Interviews zur Verfügung gestellt werden.

- Statement zur ärztlichen Vertretbarkeit

Entfällt.

10. Biometrie

- explorative Studie: Erläuterung zur statistischen Methodik:

1. Befragung der vorhandenen humanen Spendermilchbanken in Kooperation mit der FMBI: n = 30

2. Befragung der Mütter von Frühgeborenen unter 1500 Gramm über die kooperierenden Krankenkassen (DAK, TK, AOK Rheinland und pronova BKK): n = 2700

3. Befragung der ärztlichen und pflegerischen Leitungen auf deutschen neonatologischen Intensivstationen: n = 211 NICUs

4. Durchführung von Interviews mit „Experten“ im Bereich humane Spendermilchbanken: n = 6 (bzw. bis zur Sättigung)

5. Durchführung Interviews mit Müttern von Frühgeborenen zum Thema Muttermilch und Stillen: n = 12

Die Befragungen 1) 2) und 3) sind Vollerhebungen. Diese sind notwendig, um in Anbetracht des zu erwartenden Rücklaufs ausreichend Datenmaterial zu generieren um z.B. auch regionale multivariate Modelle berechnen zu können.

In Bezug auf die Stichprobengröße der qualitativen Forschung existieren, im Gegensatz zur quantitativen Forschung, keine konkreten Anhaltspunkte. Die Stichprobe ist häufig deutlich kleiner als bei der quantitativen Forschung. Dies kann zum einen durch die verfügbaren Ressourcen und zum anderen mit inhaltlichen Erwägungen begründet werden. Die Auswertung nicht-standardisierter Daten ist in der Regel deutlich aufwendiger. Eine große Stichprobe ist jedoch auch aus methodischen Gesichtspunkten nicht erforderlich, da bereits mit wenigen ProbandInnen ein tiefgehendes Spektrum an Informationen über den Gegenstandsbereich erreicht werden kann (Ziel der theoretischen Verallgemeinerung der Stichprobenergebnisse). Ausgehend von der Annahme einer theoretischen Sättigung, würden sich nur noch redundante Informationen aus einem Mehr an Interviews ergeben. Insgesamt können somit in der qualitativen Forschung keine konkreten Vorgaben für die Festlegung der Stichprobengröße gegeben werden. Es existieren jedoch Empfehlungen für die Forschungspraxis. Zunächst müssen dabei die verfügbaren Ressourcen betrachtet werden, da die Durchführung qualitativer Interviews sehr zeit- und kostenintensiv ist. Darüber hinaus ist die Reichweite der Fragestellung von Bedeutung. Die sogenannte theoretische Sättigung tritt eher ein, desto spezifischer die Fragestellung ist [1].

Insgesamt wird in der Forschungsliteratur bei qualitativen Interviews, in Abhängigkeit von der Auswertungsmethode, die Durchführung von 5 bis hin zu 50 Interviews vorgeschlagen [2]. Bezüglich der Auswertung von Interviews mithilfe der qualitativen Inhaltsanalyse hat eine Studie von Guest et al. gezeigt, dass bereits nach 6 Interviews die meisten vergebenen Kategoriencodes abgedeckt waren [3]. Dies verdeutlicht, dass die wichtigen Themen des Untersuchungsgegenstandes sich auch bereits mit sehr kleinen Stichprobengrößen abdecken lassen [1]. Die in der vorliegenden Studie gewählten Fallzahlen für die Probanden kann somit nach dem Stand der Wissenschaft begründet werden.

1. Akremi L: Stichprobenziehung in der qualitativen Sozialforschung. In: Baur N, Blasius J (eds.): Handbuch der Methoden der empirischen Sozialforschung. Wiesbaden: Springer Fachmedien 2014; 265–282.

2. Mason M: Sample Size and Saturation in PhD Studies Using Qualitative Interviews. Forum: Qualitative Sozialforschung 2010; 11(3).

3. Guest G, Bunce A, Johnson L: How Many Interviews Are Enough? Field Methods 2006; 18(1): 59–82.

11. Datenmanagement und Datenschutz

- Verantwortliche/r für die Datenverarbeitung; Datenschutzbeauftragte/r von Initiator und Studienzentrum[1]

Studienleitung Dr. Nadine Scholten, Datentreuhänder: Markus Alich (Care Research Lab)

1.: Befragung bestehende Spendermilchbanken: Pseudonyme Befragung unter Einholen einer Einverständniserklärung (EV). Die Pseudonymisierungsliste wird durch einen nicht im Projekt eingebundenen Mitarbeiter des CareResearch Labs (Datentreuhänder: Markus Alich) im IMVR verwaltet. Die Projektmitarbeiter haben hierauf keinen Zugriff. Vor der Datenanalyse durch die Wissenschaftler des Projekts findet eine Zweitpseudonymisierung durch den Datentreuhänder statt.

Rechte auf Auskunft (einschließlich Recht auf kostenfreie Kopie der Daten), Berichtigung, Löschung und Einschränkung der Datenverarbeitung nach der DSGVO werden eingeräumt. Die Einverständniserklärung kann jederzeit widerrufen werden, was zu einem Löschen der Daten führt.

Mit der EV wird gleichzeitig die Bereitschaft zur Teilnahme an einem Interview abgefragt.

2.: Befragung von Müttern von Frühgeborenen: Die Befragung findet anonym statt, wobei die vom IMVR erstellten Befragungsunterlagen durch die beteiligten Krankenkassen an Mütter von Frühgeborenen mit einem Geburtsgewicht unter 1.500 Gramm versendet werden. Das IMVR erhält durch die Kasse keine Kenntnis über direkt personenidentifizierende Angaben. Die Mütter senden dem IMVR den anonymen Fragebogen zurück, wobei bei der Erstellung der Fragebögen darauf geachtet wird, keine direkt personenidentifizierenden Angaben zu erheben. Über den Freitexten wird darauf hingewiesen, dass keine Namen genannt werden sollen. Um den teilnehmenden Müttern den Gutschein zukommen zu lassen, liegt ein Formular zur Übermittlung der E-Mail Adresse bei. Dieses ist in einem verschlossenen Umschlag dem Fragebogen beizulegen. Der Briefumschlag wir in der Vertrauensstelle geöffnet und getrennt von dem Fragebogen weiterverarbeitet. Einen Verknüpfung von Fragebogen und E-Mail Adresse findet nicht statt. Die E-Mail Adresse wird nur genutzt um den Gutscheincode zu übermitteln.

---

[1] Verantwortlicher für die Datenverarbeitung ist zumindest auch der Initiator (Sponsor oder sonstiger Träger), weil dieser – gegebenenfalls gemeinsam mit anderen – über Zwecke und Mittel der Datenverarbeitung entscheidet (vgl. die Definition in Art. 4 Nr. 7 DSGVO). Deshalb ist hier zunächst der Initiator und dessen Datenschutzbeauftragter zu nennen. Daneben kommt auch eine Verantwortlichkeit des lokalen Studienzentrums in Betracht. Es ist stets empfehlenswert, für die Wahrnehmung der Rechte nach der DSGVO (Auskunft, Löschung etc.) im Studienzentrum einen Ansprechpartner zu benennen, damit nicht zwingend der Initiator kontaktiert werden muss, der keinen Zugang zu den personenbezogenen Daten hat.

3. Befragung der ärztlichen und pflegerischen Leitungen auf deutschen neonatologischen Intensivstationen: Die Befragung findet anonym unter Rücklaufkontrolle statt.

Bei der Erstellung der Fragebögen wird darauf geachtet, dass keine direkt personenidentifizierenden Angaben erhoben werden. Über den Freitexten wird darauf hingewiesen, dass keine Namen genannt werden sollen. Die Befragung wird in Anlehnung an die Total-Design-Method nach Dillman, mit bis zu vier postalischen Erhebungswellen, durchgeführt. Die Rekrutierung erfolgt über öffentlich verfügbare Kontaktdaten. Folgendes Vorgehen ist geplant: Die ärztlichen und pflegerischen Leitungspersonen erhalten das Anschreiben und den Fragebogen und werden darüber aufgeklärt, dass Ihre Teilnahme an der Befragung als implizite Einverständniserklärung zum Verfahren laut Studieninformation gewertet wird, die Befragung selbst daher anonym stattfindet. Die frankierten Rückumschläge der Leitungspersonen sind außen mit einer Nummer versehen, sodass im IMVR eine Kontrolle des Rücklaufs gewährleistet werden kann. Dies geschieht folgendermaßen: 1.) Die Nummern der im IMVR eingehenden Umschläge werden von der ursprünglichen Adressliste gelöscht 2.) Erinnerungen und damit der wiederholte Fragebogen-Versand erfolgen auf diese Weise nur noch auf Basis der verbleibenden Adressen. 3.) Nach Abschluss der (bis zu) vier Erhebungswellen wird die Adressliste gelöscht und erst danach erfolgt 4.) die Öffnung der Umschläge und die Auswertung der anonymen Fragebogen. Alle eingehenden Umschläge werden bis dahin in einem abgeschlossenen Lagerraum im IMVR aufbewahrt. Mithilfe dieses Verfahrens sind zielgerichtete Erinnerungen möglich und zugleich wird vermieden, diejenigen Leitungspersonen, die bereits teilgenommen haben, mehrfach anzuschreiben und ggf. zu bedrängen. Oberstes Ziel bei dem gewählten Verfahren ist eine möglichst hohe Beteiligung an der Befragung unter Wahrung der Anonymität. Ein Vorgehen ohne Rücklaufkontrolle und gezielte Erinnerungswellen gefährdet den zu erzielenden Rücklauf.

4. Durchführung von Interviews mit Experten im Bereich humane Spendermilchbanken und 5. Durchführung Interviews mit Müttern von Frühgeborenen zum Thema Muttermilch und Stillen: Zu Auswertungszwecken werden während der Interviews, nach Aufklärung und Einverständnis der TeilnehmerInnen, Audioaufnahmen gemacht. Die Aufnahmen werden pseudonymisiert und für die Transkription an das externe Dienstleistungsunternehmen übergeben. Dazu wird mit dem Dienstleister ein Vertrag zur Einhaltung des Datenschutzes geschlossen. Für die Auswertungen genutzt werden nur die anonymen Transkripte.

Rechte auf Auskunft (einschließlich Recht auf kostenfreie Kopie der Daten), Berichtigung, Löschung und Einschränkung der Datenverarbeitung nach der DSGVO wird eingeräumt. Die Einverständniserklärung kann jederzeit widerrufen werden, was zu einem Löschen der Daten führt.

12. Unterschriften: Studienleiter/in (Antragsteller/in)

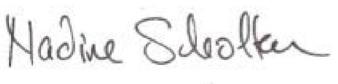

Anhang: Liste der beteiligten Studienzentren (bei multizentrischen Studien)

Universität Bielefeld:

Prof. Dr. Friederike Eyssel	CITEC Center of Excellence Cognitive Interaction Technology, Universität Bielefeld	Tel.: +49521 106 12044 feyssel@cit-ec.uni-bielefeld.de	AP 3: Konzept zur Stillförderung
